# Allele-Specific Induction of IL-1β Expression by C/EBPβ and PU.1 Contributes to Increased Tuberculosis Susceptibility

**DOI:** 10.1371/journal.ppat.1004426

**Published:** 2014-10-16

**Authors:** Guoliang Zhang, Boping Zhou, Shaoyuan Li, Jun Yue, Hui Yang, Yuxin Wen, Senlin Zhan, Wenfei Wang, Mingfeng Liao, Mingxia Zhang, Gucheng Zeng, Carl G. Feng, Christopher M. Sassetti, Xinchun Chen

**Affiliations:** 1 Guangdong Key Lab of Emerging Infectious Diseases, Guangdong Medical College, Shenzhen, China; 2 Shenzhen Key Lab of Infection and Immunity, Shenzhen Third People's Hospital, Guangdong Medical College, Shenzhen, China; 3 Department of Microbiology, Key Laboratory for Tropical Diseases Control of the Ministry of Education, Zhongshan School of Medicine, Sun Yat-sen University, Guangzhou, China; 4 Department of Clinical Laboratory, Shanghai Pulmonary Hospital, Tongji University, Shanghai, China; 5 Department of Chest Surgery, Shenzhen People's Hospital, Jinan University, Shenzhen, China; 6 Department of Infectious Diseases and Immunology, Sydney Medical School, The University of Sydney, Sydney, Australia; 7 Department of Microbiology and Physiological Systems, University of Massachusetts Medical School, Worcester, Massachusetts, United States of America; 8 Howard Hughes Medical Institute, Chevy Chase, Maryland, United States of America; University of Washington, United States of America

## Abstract

*Mycobacterium tuberculosis* infection is associated with a spectrum of clinical outcomes, from long-term latent infection to different manifestations of progressive disease. Pro-inflammatory pathways, such as those controlled by IL-1β, have the contrasting potential both to prevent disease by restricting bacterial replication, and to promote disease by inflicting tissue damage. Thus, the ultimate contribution of individual inflammatory pathways to the outcome of *M. tuberculosis* infection remains ambiguous. In this study, we identified a naturally-occurring polymorphism in the human *IL1B* promoter region, which alters the association of the C/EBPβ and PU.1 transcription factors and controls Mtb-induced IL-1β production. The high-IL-1β expressing genotype was associated with the development of active tuberculosis, the severity of pulmonary disease and poor treatment outcome in TB patients. Higher IL-1β expression did not suppress the activity of IFN-γ-producing T cells, but instead correlated with neutrophil accumulation in the lung. These observations support a specific role for IL-1β and granulocytic inflammation as a driver of TB disease progression in humans, and suggest novel strategies for the prevention and treatment of tuberculosis.

## Author Summary

IL-1β is important for the initial establishment of antimicrobial adaptive immunity, but prolonged IL-1β expression can also cause progressive immunopathology during M. tuberculosis infection. The paradoxical activities of IL-1β in promoting both antimycobacterial immunity and chronic tissue damage have left the ultimate contribution of this cytokine to TB progression in human populations unclear. In this work, we address the role of IL-1β-mediated inflammation using a combination of human genetics and molecular biology, and suggest that exuberant IL-1β responses are causatively associated with TB progression and poor treatment outcome in humans. This work furthers our understanding of the immunological factors that underlie TB disease and provide a strong rationale for the development of specific anti-inflammatory adjunctive therapies that could improve the long-term outcome of TB treatment. In addition, these insights inform the design of future TB control efforts that include the rational design of disease-preventing vaccines and genotype-targeted delivery of TB chemotherapy.

## Introduction

Tuberculosis (TB), a chronic bacterial disease caused by *Mycobacterium tuberculosis* (Mtb), remains a major global health problem that claims 1.4 million lives annually. Natural infection with Mtb is initiated by the deposition of Mtb-containing aerosol droplets onto lung alveolar surfaces, and infection at this site can produce a wide spectrum of clinical outcomes. The vast majority of immunocompetent individuals contain the pathogen and remain indefinitely asymptomatic, a status defined as “latent” TB [1]. A smaller proportion of Mtb-infected people develop active disease, most often characterized by a progressive inflammatory pathology of the lung. The hematogenous dissemination of Mtb can also result in disease at a variety of extra pulmonary sites, commonly including bone and the central nervous system [2].

The mechanisms controlling TB progression remain elusive. Both Mendelian genetic susceptibilities in the IL-12 and IFN-γ axis [3], [4], and the association of TB with HIV-mediated lymphocyte depletion indicate that cell-mediated adaptive immunity is critical for controlling mycobacterial growth and containing disease. However, genetic association studies also commonly identify single nucleotide polymorphisms (SNPs) in pro-inflammatory innate immune mediators that are associated with TB susceptibility and regulate cellular function, such as TLRs, LTA4H, IL22, and IL6 [5], [6], [7], [8], [9], [10]. These associations suggest that disease progression may be determined at multiple levels in human populations and that the inflammatory response could play a decisive role.

As a potent proinflammatory cytokine, IL-1β plays an important role in many inflammation-related diseases as well as cancer. Accordingly, *IL1B* gene polymorphisms, especially functional SNPs -31 T>C (rs1143627) and -511 G>A (rs16944) are associated with susceptibility to a number of diseases [11], [12], [13], [14]. IL-1β is also important in the pathogenesis of TB in mice [15], and polymorphisms in the human *IL1B* gene have been suggested to have differing effects on TB susceptibility. Awomoyi reported the *IL1B* rs16944, but not +3953 T>C (rs1143634) SNP, was associated with susceptibility to TB in a Gambian population [16]. Kusuhara et al reported that three SNPs in *IL1B* (rs1143629, rs1143643 and rs3917368) are associated with TB susceptibility in a Japanese population [17]. Similarly, susceptibility to TB is associated with the polymorphic +3953 region in the *IL1B* gene [18]. Using an oligochip-based method, Sun et al genotyped two SNPs (rs1143627, rs16944) in a small cohort of the Chinese Han population (98 TB patients and 65 healthy controls), and found that the rs1143627T allele was associated with TB susceptibility [19]. On the contrary, a strong protection conferred by *IL1B* +3953 T-allele-carrying genotypes was observed in a Northwestern Colombian population [20]. While these studies implicated several SNPs in *IL1B* gene were associated with TB susceptibility, a conclusive role of *IL1B* SNPs has not yet been established due to the small sample sizes and different ethnic background in these studies. Furthermore, the mechanisms by which these polymorphisms might act remain unclear.

Recent mechanistic studies in the mouse model have implicated IL-1β mediated granulocytic inflammation as a potential driver of TB progression [21], [22], [23]. IL-1β is a prototypical proinflammatory cytokine that stimulates both local and systemic responses [24], and this cytokine plays a complex dual role in chronic infections. The production of IL-1β is important for the proper development of antimicrobial adaptive immunity during the initial stages of infection [25], [26]. However, prolonged IL-1β production promotes the continual recruitment of granulocytes to the lung, induces the expression of additional inflammatory mediators such as prostaglandin E2, and stimulates tissue-damaging metalloproteinases [27], [28]. As a result, the production of this cytokine must be repressed in chronically Mtb-infected animals to avoid progressive pathology [21], [29]. While, production of IL-1β correlates with the severity of human TB disease [30], [31], its paradoxical activities in promoting both antimycobacterial immunity and chronic tissue damage leave the ultimate contribution of this cytokine to TB progression in human populations unclear.

In this study, we identified a genetic polymorphism (rs1143627) in the promoter region of the *IL1B* gene that increases the C/EBPβ- and PU.1-dependent expression of the cytokine. The high-IL-1β-expressing genotype is associated with increased risk of active tuberculosis and poor clinical outcome. The observed correlation between IL-1β production, neutrophil recruitment, and TB susceptibility indicates a causative role for IL-1β-mediated granulocytic inflammation in TB progression.

## Results

### *IL1B* promoter polymorphism is associated with susceptibility to tuberculosis

Genetic variants in the *IL1B* gene, especially those in the promoter region, can affect cytokine expression and have been associated with susceptibility to inflammatory disorders [24], [32] and chronic infections [33], [34]. Since TB is a disease of chronic inflammation, we hypothesized that similar genetic variants might alter susceptibility to this disease. To test our hypothesis, the genotype distribution of 4 *IL1B* SNPs with potential regulatory effects was first determined in healthy controls and TB patients from a cohort in Shenzhen and then replicated in a Shanghai cohort (Table 1). Among them, the following three SNPs were located within the known promoter region, -31 T>C (rs1143627), -511 G>A (rs16944), and -1473 G>C (rs1143623). The final SNP, +7326 T>C (rs2853550), is located in the 3-UTR of *IL1B* gene. The minor allele frequencies of all 4 SNPs were>10% and were in Hardy-Weinberg equilibrium in both control groups (*P*>0.05). However, only the frequency of rs1143627 was significantly different between patients with active TB (TB, n = 1533) and healthy controls (HC, n = 1445) in the Shenzhen cohort. Specifically, a significantly higher frequency of the T allele at rs1143627 was observed among patients with active TB, compared with controls, indicating that this allele is associated with an increased risk of tuberculosis (odds ratio [OR] = 1.20; 95% confidence interval [CI], 1.09–1.33; *P* = 0.0004). At the genotype level, carriage of the *IL1B* rs1143627T allele increased the apparent risk of active TB (OR = 1.35; 95% CI, 1.14–1.59; *P* = 0.0005, dominant model). TT and TC genotypes also showed increased TB susceptibility compared to CC genotype using an additive model (OR = 1.44; 95%CI, 1.18–1.76; *P* = 0.0004; and OR = 1.30; 95%CI, 1.09–1.55; *P* = 0.004) (Table 2).

**Table 1:** Characteristics of patients with active TB and healthy controls in multiple cohorts.

Cohort	Subgroup	No.	Age, Years (mean±SD)	Sex (Male∶Female)
Shenzhen cohort				
Healthy controls	—	1445	36.84±19.71	882∶563
Active TB	Pulmonary TB	1432	37.08±14.17	920∶512
	Extrapulmonary TB^a^	101	34.69±12.68	60∶41
Shanghai cohort				
Healthy controls	—	262	39.84±10.43	140∶122
Active TB	Pulmonary TB	266	41.72±14.05	147∶119

a Including tuberculous lymphadenitis (n = 62), tuberculous meningitis (n = 13), and osteoarticular tuberculosis (n = 26)

**Table 2:** Association between *IL1B* Gene SNPs and TB susceptibility in Shenzhen cohort.

		HC	TB	Multiplicative		Additive		Dominant		Recessive	
SNP ID	Genotype	No (%)	No (%)	P Value	OR (95% CI)	P Value	OR (95% CI)	P Value	OR (95% CI)	P Value	OR (95% CI)
rs1143627	TT	357 (24.7)	436 (28.4)	0.0004	1.20(1.09–1.33)	0.0004	1.44(1.18–1.76)	0.0005	1.35(1.14–1.59)	0.02	0.83(0.70–0.97)
	TC	693 (48.0)	762 (49.7)			0.004	1.30(1.09–1.55)				
	CC	395 (27.3)	335 (21.8)			Ref.	Ref.				
rs1143623	GG	520 (36.0)	511 (33.3)	0.673	0.98(0.88–1.09)	0.814	1.03(0.82–1.28)	0.232	1.13(0.93–1.38)	0.128	0.89(0.76–1.03)
	CG	692 (47.9)	799 (52.1)			0.080	1.21(0.98–1.49)				
	CC	233 (16.1)	223 (14.5)			Ref.	Ref.				
rs16944	GG	408 (28.2)	404 (26.4)	0.682	0.98(0.88–1.08)	0.733	0.97(0.79–1.18)	0.601	1.05(0.88–1.24)	0.250	0.91(0.77–1.07)
	AG	683 (47.3)	766 (50.0)			0.327	1.09(0.91–1.31)				
	AA	354 (24.5)	363 (23.7)			Ref.	Ref.				
rs2853550	TT	620 (42.9)	642 (41.9)	0.508	0.96(0.87–1.07)	0.541	0.93(0.74–1.17)	0.623	0.95(0.77–1.17)	0.571	0.96(0.83–1.11)
	TC	635 (43.9)	680 (44.4)			0.752	0.96(0.77–1.21)				
	CC	190 (13.1)	211 (13.8)			Ref.	Ref.				

Note: Hardy-Weinberg equilibrium P values of 4 SNPs (in the order of rs1143627, rs1143623, rs16944, rs2853550) were 0.12, 0.91, 0.06, and 0.17 in HC, and were 0.95, 0.01, 0.99, and 0.15 in TB.

The association of rs1143627 with tuberculosis was replicated in an independent Shanghai cohort. In this group the T allele was associated with TB susceptibility using a multiplicative model, with an adjusted OR of 1.40 (95% CI, 1.10–1.77, P = 0.007). Using an additive model the T allele was estimated to impart a 97% increase in risk (OR = 1.97; 95% CI, 1.20–3.23; P = 0.006) (Table 3). These observations from two large independent cohorts were similar and consistent with the reported distribution of rs1143627 and rs16944 alleles in separate small cohort of Chinese TB patients and healthy controls [35]. When we pooled the Shenzhen and shanghai data together (1799 cases and 1707 controls) for statistical analysis, the effect of rs1143627T was even stronger in all genetic models (multiplicative, additive, dominant and recessive, Table S1) than in either cohort alone. Thus, we conclude that the T allele of *IL1B* SNP rs1143627 is associated with susceptibility to active TB in the Chinese population.

**Table 3:** Replication of association between rs1143627 SNP and TB susceptibility in Shanghai cohort.

		HC	TB	Multiplicative		Additive		Dominant		Recessive	
SNP ID	Genotype	No (%)	No (%)	P Value	OR (95% CI)	P Value	OR (95% CI)	P Value	OR (95% CI)	P Value	OR (95% CI)
rs1143627	TT	59 (22.5)	81 (30.4)	0.007	1.40(1.10–1.77)	0.006	1.97(1.20–3.23)	0.018	1.63(1.08–2.46)	0.039	1.50(1.02–2.22)
	TC	131 (50.0)	135 (50.7)			0.07	1.48(0.96–2.29)				
	CC	72 (27.5)	50 (18.8)			Ref.	Ref.				

Note: Hardy-Weinberg equilibrium P values of rs1143627 were 0.96 and 0.63 in HC and TB respectively.

While TB is primarily a disease of the lung, dissemination of the bacterium can also result in pathology at a variety of different tissue sites. To understand if the rs1143627 SNP differentially affected pulmonary versus extrapulmonary disease, we further investigated whether the distribution of rs1143627 alleles differed in patients suffering from distinct manifestations of TB. As shown in Table 4, the rs1143627T allele is significantly associated with both pulmonary TB (PTB, OR = 1.17; 95% CI, 1.06–1.30; *P* = 0.003) as well as extrapulmonary disease (ETB, OR = 1.78; 95% CI, 1.32–2.39; *P*<0.0001) using a multiplicative model. However, the frequency of the rs1143627T allele was significantly higher in patients with extrapulmonary TB than in those with pulmonary disease (*P* = 0.005). Thus, among TB patients, individuals carrying rs1143627T allele are more prone to develop extrapulmonary TB than those without (OR = 1.53, 95% CI, 1.14–2.05, multiplicative model).

**Table 4:** Association between rs1143627 SNP and susceptibility to various clinical phenotypes of TB in Shenzhen cohort.

		HC	PTB	ETB	PTB Vs HC		ETB Vs HC		ETB Vs PTB	
SNP ID	Genotype	No (%)	No (%)	No (%)	P Value	OR (95%CI)	P Value	OR (95%CI)	P Value	OR (95%CI)
**rs1143627**	TT	357 (24.7)	395 (27.6)	41 (40.5)	0.003	1.17(1.06–1.30)	<0.0001	1.78(1.32–2.39)	0.005	1.53(1.14–2.05)
	TC	693 (48.0)	717 (50.1)	45 (44.6)						
	CC	395 (27.3)	320 (22.3)	15 (14.9)						

### *IL1B* polymorphism is associated with more severe TB disease

**Figure 1:**
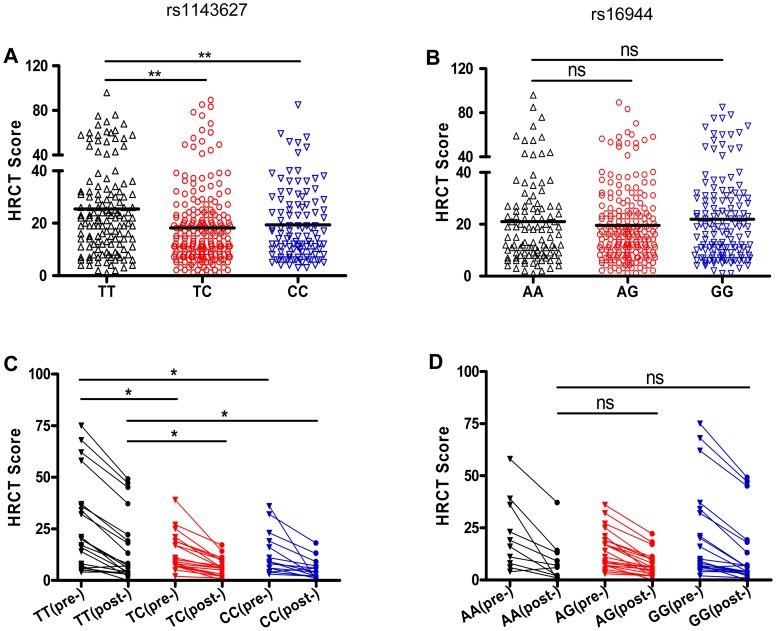
SNP rs1143627 is associated with the severity of pulmonary TB disease. (A and B) The HRCT scores were determined in a total of 453 patients with pulmonary TB before initiation of anti-TB chemotherapy. The HRCT scores in patients carrying different rs1143627 genotypes (A) or rs16944 genotypes (B) were compared. (C and D) The HRCT scores were determined in 53 of 453 patients who have performed HRCT examinations before and 2 years after completion of anti-TB chemotherapy. The HRCT scores [before (pre-) and 2 years after (post-) initiation of treatment] in patients carrying different rs1143627 genotypes (C) or rs16944 genotypes (D) were compared. Differences between groups were compared with the ANOVA/Newman-Keuls multiple comparison test. *, p<0.05; **, p<0.01; ns, not significant.

The proinflammatory activity of IL-1β suggested that the rs1143627 polymorphism might affect the inflammatory response to Mtb, which could be exhibited at either systemic or local levels. No difference in erythrocyte sedimentation rate (ESR) or C-reactive protein (CRP) levels were apparent among pulmonary TB patients carrying different rs1143627 genotypes (TT, TC, CC) (Fig. S1). However, using high-resolution computed tomography (HRCT), we were able to directly quantify lung damage in these patients using a score based on radiographic manifestations including the presence of nodules, cavities, and bronchial lesions [36], [37]. A total of 453 out of 1432 patients with primary pulmonary TB received an HRCT score before anti-TB treatment. Among these individuals, the HRCT scores were significantly higher in patients carrying rs1143627TT genotype than those with rs1143627CC genotype (Fig. 1A). Even 2 years after the completion of anti-TB treatment, patients (n = 53) of the rs1143627TT genotype still displayed significantly higher HRCT scores than those carrying rs1143627CC, suggesting that the rs1143627 polymorphism is associated with long-term lung damage and disease outcome (Fig. 1C). In contrast, no difference of HRCT score was found in patients carrying different genotypes of rs16944 (Fig. 1B and D), an *IL1B* SNP that is associated with the risk of developing active TB in the Gambian population [16], but not in our current study. Taken together, these results indicated that the rs1143627T allele is associated with more severe pulmonary TB and the expression of extrapulmonary disease.

### The rs1143627T allele increases IL-1β production, but not IL-Ra expression

**Figure 2:**
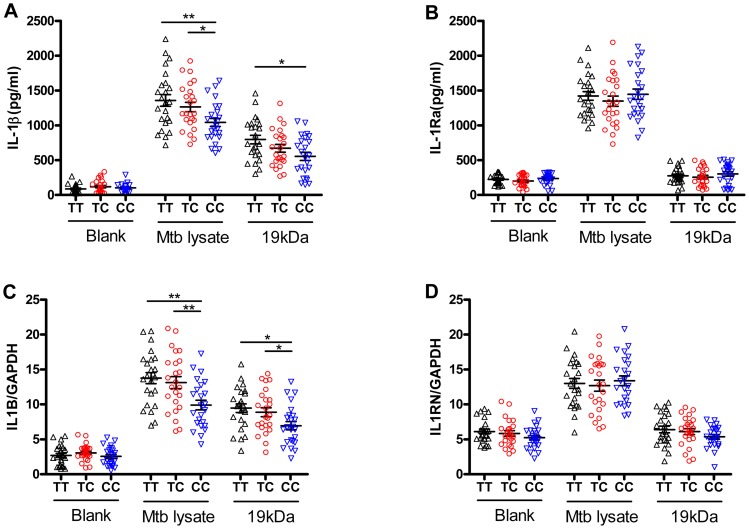
The rs1143627 polymorphism affects IL-1β production by monocytes upon Mtb stimulation. CD14^+^ monocytes were isolated from PBMCs of healthy controls carrying different rs1143627 genotypes (TT, n = 24; TC, n = 24; CC, n = 24). Purified CD14^+^ monocytes were cultured in the absence or presence of heat-killed Mtb lysate (20 µg/mL) or Mtb 19 kDa lipoprotein (0.5 µg/mL,). (A and B) The concentrations of IL-1β (A) and IL-Ra (B) in the culture supernatant were determined by ELISA after incubation for 24 h. (C and D) The *IL1B* (C) and *IL1RN* (D) mRNA levels were determined using SYBR Green-based real-time qPCR after incubation for 24 h, and the data was expressed as mRNA copy number relative to house keeping gene *GAPDH*. Differences between groups were compared with the ANOVA/Newman-Keuls multiple comparison test. *, p<0.05; **, p<0.01.

Located at position -31 from the transcriptional start site, SNP rs1143627 is in the promoter region of *IL1B* gene. Previous reports indicated that rs1143627 affects transcription of IL-1β in response to lipopolysaccharide [38]. To determine whether this polymorphism affects gene expression in response to Mtb stimulation, we assessed IL-1β expression in CD14+ monocytes isolated from healthy controls carrying different rs1143627 genotypes in response to heat-killed Mtb or the 19 kDa lipoprotein derived from this bacillus. In response to both stimuli, monocytes isolated from individuals carrying rs1143627TT and TC genotypes produced significantly higher amounts of *IL1B* protein (Fig. 2A) and mRNA (Fig. 2C) than those carrying the CC genotype. Since the bioactivity of IL-1β also involves the antagonistic effects of IL-1Ra, we assessed the concentration of IL-1Ra in the culture supernatant and *IL1RN* mRNA cell lysates. As shown in Fig. 2B and D, no significant differences were observed in IL-1Ra expression upon Mtb lysate stimulation among different rs1143627 genotypes. Together, these results suggest that the rs1143627T allele specifically increased the amount of bioactive IL-1β produced by Mtb stimulation.

### The rs1143627T allele does not impair the anti-mycobacterial adaptive immune response, but promotes neutrophil infiltration to the lung

**Figure 3:**
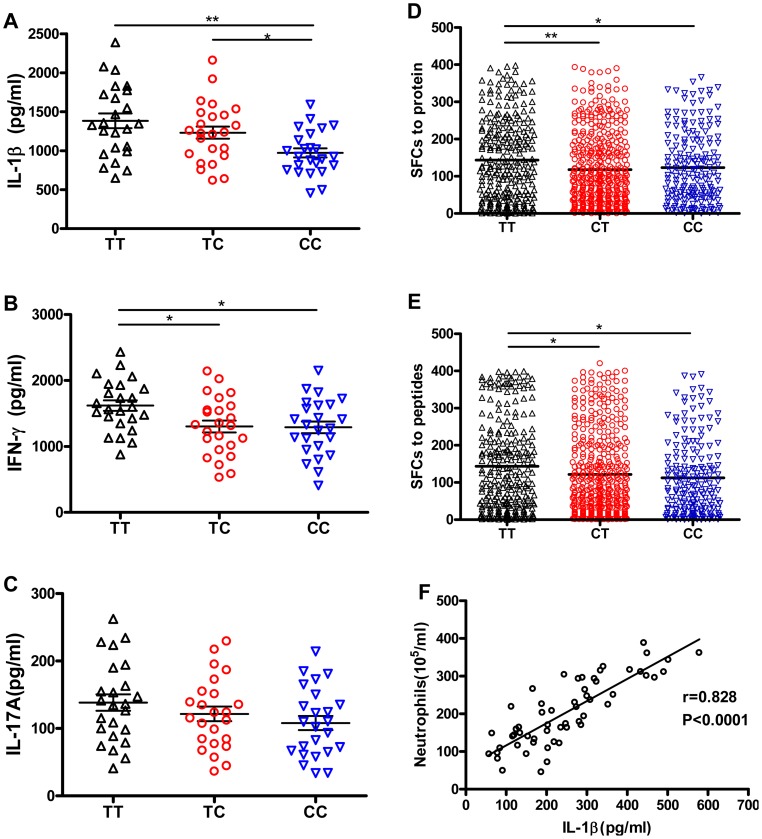
rs1143627T-induced IL-1β has little effect on Mtb-specific lymphocyte responses, but IL-1β correlates with neutrophil recruitment to the lung. PBMCs from healthy controls carrying different rs1143627 genotypes (TT, n = 24; TC, n = 24; CC, n = 24) were cultured in the absence of Mtb lysate (20 µg/mL) for 48 h. (A-C) The concentrations of secreted IL-1β (A), IFN-γ (B) and IL-17A (C) were determined by ELISA. (D and E) Mtb antigen (ESAT-6 protein, or ESAT-6/CFP-10 peptide pool) specific IFN-γ production by PBMCs from patients with PTB carrying different rs1143627 genotypes was quantified by ELISPOT assay. Data were expressed as the number of IFN-γ SFCs per 2×10^5^ PBMCs of each subjects. Differences between groups were compared with the ANOVA/Newman-Keuls multiple comparison test. (F) Correlation analysis between the levels of IL-1β and the number of neutrophils in the BALF. The coefficient r and *P* values are indicated. *, p<0.05; **, p<0.01.

IL-1β is recognized to play an important role in shaping adaptive immunity, especially Th17 cell responses [39], [40]. To understand if the rs1143627T allele could promote TB disease by inhibiting the expression of adaptive immunity, we investigated whether this polymorphism altered the production of Mtb antigen-specific IFN-γ or IL-17A, the hallmark cytokines of Th1 and Th17 cells, respectively. PBMC isolated from healthy controls carrying different rs1143627 genotypes were cultured in the presence of heat-killed Mtb and the production of IFN-γ and IL-17A were compared. Consistent with our previous results with purified monocytes, we found that PBMC from individuals carrying rs1143627 TT and TC genotypes produced more IL-1β than those from individuals carrying the CC genotype (Fig. 3A). The rs1143627TT PBMC also produced slightly higher levels of IFN-γ and similar levels of IL-17A as rs1143627 CC cells (Fig. 3, B and C). The increased production of IFN-γ was a result of IL-1β secretion, as IFN-γ and IL-1β secretion were highly correlated in PBMC cultures and IFN-γ production could be inhibited by the addition of a blocking antibody to IL-1β (Fig. S2).

The effect of rs1143627 on the production of IFN-γ-producing cells was further investigated in a large cohort of pulmonary TB patients. Mtb antigen-specific IFN-γ production by PBMCs was detected by using a previously established IFN-γ Elispot assay that employs either ESAT-6 protein or an ESAT-6/CFP-10 peptide pool as a stimulant [41], [42]. Patients carrying the rs1143627TT genotype had significantly higher numbers of Mtb antigen-specific IFN-γ spot forming cells (SFCs) than those carrying the CC genotype (Fig. 3, D and E). In contrast, the numbers of IFN-γ SFCs were not different among patients carrying different rs16944 genotypes (Fig. S3). Thus, the TB-associated rs1143627T allele was associated with an augmentation of the canonical antimycobacterial response. We conclude that the susceptibility of individuals carrying the rs1143627T allele is unlikely to be the result of a failure to respond to mycobacterial antigen.

Another primary effect of IL-1β is the recruitment of neutrophils, a process that can exacerbate TB pathogenesis in animal models [21], [22], [23]. To investigate whether this cytokine could play a similar role in human TB, we simultaneously measured the level of IL-1β and the number of neutrophils in broncheoalveolar lavage fluid (BALF) from patients with active pulmonary TB. A significant correlation between the level of IL-1β and the number of recruited neutrophils was found (r = 0.828, *P*<0.0001) (Fig. 3F). Thus, the increased production of IL-1β associated with the rs1143627T allele could promote lung damage and TB progression by stimulating granulocytic inflammation.

### SNP rs1143627 influences C/EBPβ and PU.1 binding to *IL1B* promoter

**Figure 4:**
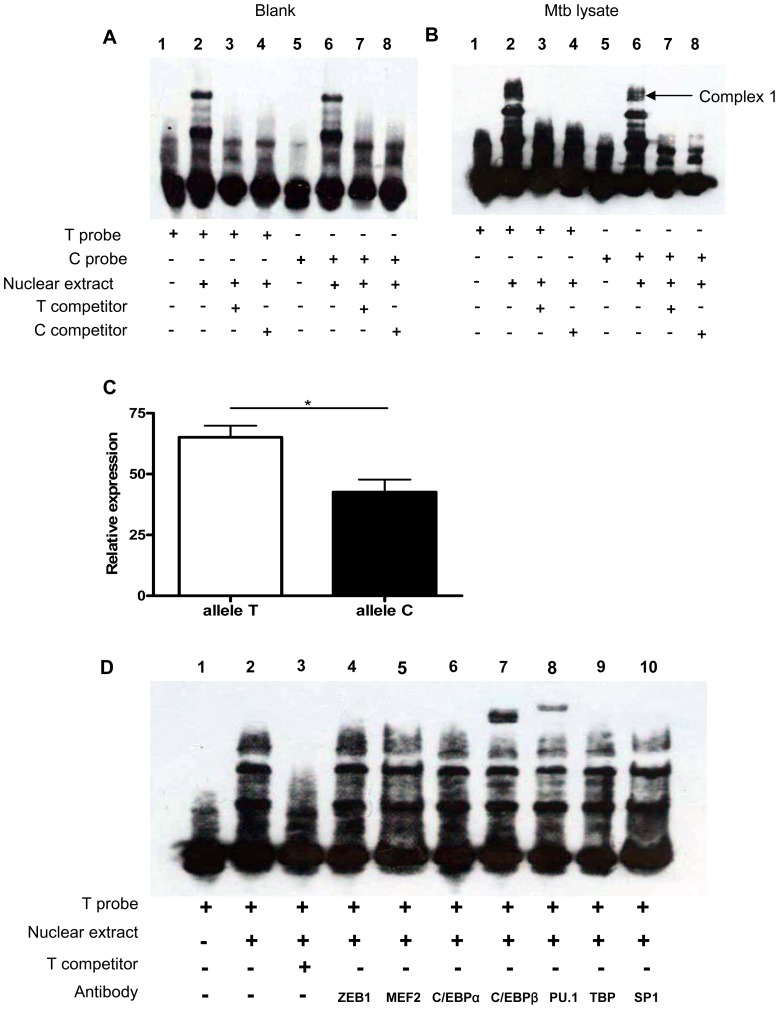
SNP rs1143627 variant affects C/EBPβ and PU.1 binding to the *IL1B* promoter. (A and B) Nuclear extracts were prepared PMA-differentiated U937 cells stimulated without (A) or with (B) Mtb lysate (20 µg/mL) for 24 h. Nuclear extracts were incubated with T-probe (lanes 1–4) or C-probe (lanes 5–8) in the absence or presence of unlabelled competitors and subjected to EMSA. (C) The relative abundance of complex 1 bound to T probe or C probe in (B) quantified by densitometry. Data are shown as the mean ± SEM, and differences between groups were compared with unpaired t tests. (D) Nuclear extracts prepared from (A) were incubated with T-probe in the absence (lane 2) or presence of antibodies against transcription factors including ZEB1, MEF2, C/EBPα, C/EBPβ, PU.1, TBP, and SP1 (lanes 4–10). Anti-C/EBPβ (lane 7) and anti-PU.1 (lane 8) supershift the DNA-protein complex 1. *, p<0.05.

The rs1143627 defines a T>C mutation at the -31 position of the *IL1Β* promoter. We used electrophoretic mobility-shift analysis (EMSA) to determine if this polymorphism altered protein binding to this promoter region. Synthetic allele-specific oligonucleotides representing the polymorphic rs1143627 sites were incubated with nuclear protein extracts from human monocytic U937 cells stimulated without or with killed Mtb lysate. There was no difference in binding activity between rs1143627T oligonucleotide (T Probe) and rs1143627C oligonucleotide (C Probe) when U937 were not stimulated with Mtb lysate (Fig. 4A). Previous Mtb stimulation of these cells induced the formation of a DNA binding complex on the rs1143627T oligonucleotide (“*complex 1*”, Figure 4B). In contrast, Mtb exposure induced less *complex 1* formation on the rs1143627C oligonucleotide (Fig. 4B, lane 2 and 6; Fig. 4C). Furthermore, *complex 1* formation on radiolabelled rs1143627 was specifically blocked by competition with the unlabelled oligonucleotide (Fig. 4B, lanes 3, 4, 7, and 8). These results indicated that the binding of one or more proteins to the *IL1B* promoter region was altered by rs1143627 polymorphism, which could cause the observed difference in IL-1β expression that was associated with these genotypes.

Bioinformatics-based prediction analysis using alibaba and match algorithm (http://www.gene-regulation.com) and previous studies of the *IL1B* promoter region indicated that transcription factors C/EBPa, C/EBPβ, PU.1, TBP and SP1 are involved in the regulation of *IL1B* gene expression and have the potential to bind in the polymorphic region [43], [44]. To determine if these proteins were part of the rs1143627-modulated *complex 1, we* conducted supershift experiments using antibodies against each transcription factor. Addition of antibodies against C/EBPβ and PU.1, but not antibodies against RSRFC4, C/EBPa, TBP or SP1, to the rs1143627T oligonucleotide binding reactions resulted in shift of complex 1 to a higher molecular weight species (Fig. 4D). Thus, the protein complex formed on the rs1143627-containing region includes PU.1 and C/EBPβ, suggesting that the T>C polymorphism could alter IL-1β expression by influencing the binding of transcription complexes that contain these factors.

### Expression of PU.1 and C/EBPβ is induced by Mtb and temporally-correlated with IL-1β expression

**Figure 5:**
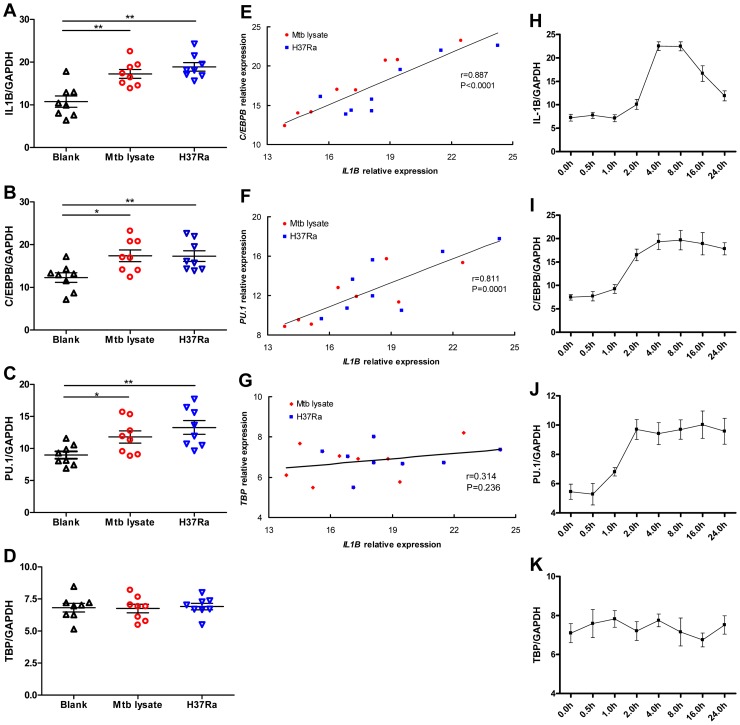
*C/EBPB* and *PU.1* expression induced by Mtb temporally correlates with *IL1B* expression. (A-D) SYBR Green-based real-time qPCR assay for the gene expression of *IL1B* (A), *C/EBPB* (B), *PU.1* (C), and *TBP* (D) by differentiated U937 cells in response to Mtb lysate (20 µg/mL) or H37Ra infection (MOI = 10). The data are expressed as mRNA copy number relative to the house keeping gene *GAPDH*. Differences between groups were compared with the ANOVA/Newman-Keuls multiple comparison test. (E-G) Correlation analysis between the *C/EBPB* (E), *PU.1* (F), or *TBP* (G) expression and *IL1B* expression. The coefficient r and *P* values are indicated. (H-K) Dynamic change of *IL1B* (H), *C/EBPB* (I), *PU.1* (J) and *TBP* (K) expression by differentiated U937 upon stimulation with Mtb lysate (20 µg/mL). *, p<0.05; **, p<0.01.

To determine if C/EBPβ and PU.1 are produced in a manner that is consistent with the proposed role in IL-1β regulation, we quantified the expression of all three genes in the monocyte-like U937 cells after stimulation with heat killed Mtb or infection with the live attenuated strain of Mtb, H37Ra. Consistent with previous reports, both stimuli induced *IL1B* mRNA expression (Fig. 5A). More importantly, we found Mtb exposure also induced the expression of *C/EBPB* (Fig. 5B) and *PU.1* (Fig. 5C), but no significant difference was observed in *TBP* expression (Fig. 5D). Furthermore, the mRNA level of *IL1B* was significantly correlated with that of both *C/EBPB* (r = 0.887, *P*<0.0001, Fig. 5E) and *PU.1* (r = 0.811, *P* = 0.0001, Fig. 5F), and not correlated with TBP expression (r = 0.314, *P* = 0.236, Fig. 5G). The induction of *PU.1* and *C/EBPB* mRNA expression occurred earlier after stimulation than *IL1B* (Fig. 5H-K), which is consistent with a role for PU.1 and C/EBPβ in regulating the *IL1B* promoter in response to Mtb infection.

### PU.1 and C/EBPβ differentially transactivate *IL1B* promoter variants determined by the rs1143627 polymorphism

**Figure 6:**
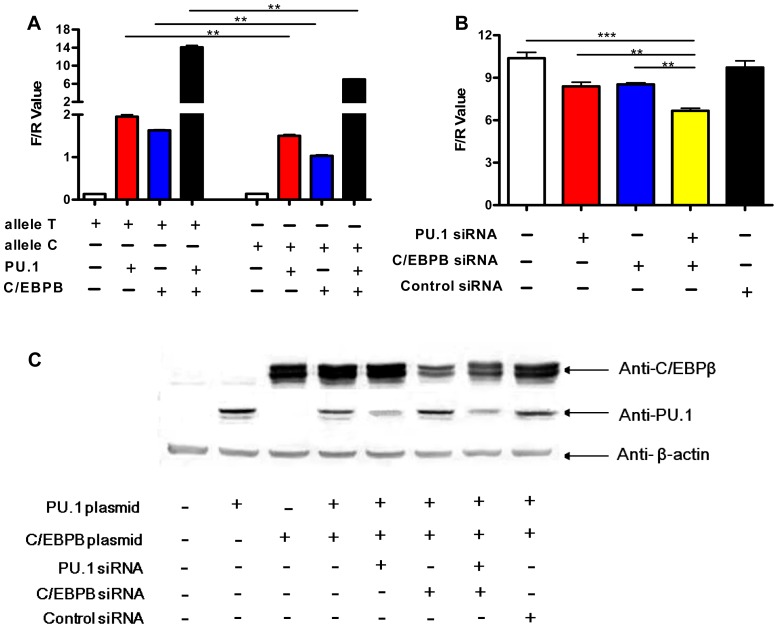
Allele-specific effects of rs1143627 are mediated through PU.1 and C/EBPβ. (A) *IL1B* promoter luciferase reporter plasmids carrying rs1143627 T or C allele were transfected into HeLa cells, with or without co-transfecting PU.1- or C/EBPβ-expressing plasmids. Luciferase activities of HeLa cells were determined and normalized to Renillla luciferase activities. (B) HeLa cells cotransfected with the T-allele *IL1B* promoter luciferase reporter plasmids, PU.1- and C/EBP-β- expressing plasmids were infected with PU.1, C/EBPβ, or control siRNA-carrying lentiviral vector constructs. Luciferase activities of HeLa cells were determined and normalized to Renillla luciferase activities. Differences between groups were compared with the ANOVA/Newman-Keuls multiple comparison test. (C) Western-blotting assay shows the expression of PU.1 or C/EBPβ after transfection or siRNA knockdown using monoclonal antibodies against PU.1 and C/EBPβ. **, p<0.01, ***, p<0.001.

To assess the role of PU.1 and C/EBPβ in Mtb-induced *IL1B* transcription, we investigated whether ectopic PU.1 and C/EBPβ expression affects *IL1B* promoter activity in HeLa cells. As shown in Fig. 6A, transfection of a reporter plasmid containing a 1371 bp fragment of the human *IL1B* promoter (−1292 to +79) produced very low luciferase levels. However, cotransfection of the *IL1B* promoter plasmid with PU.1 or C/EBPβ expression plasmids significantly enhanced luciferase levels, and cotransfection of all three plasmids further increased promoter activity (Fig. 6A). Expression levels of PU.1 and C/EBPβ were similar when transfected either individually or in combination (Fig. 6C).

siRNA knockdown studies verified the contribution PU.1 and C/EBPβ in Mtb-induced *IL1B* transcriptional activity in this transfection system. The expression of both transcription factors could be partially inhibited by siRNA transfection (Fig. 6C). Knockdown of either protein significantly inhibited the Mtb-induced *IL1B* promoter activity (Fig. 6B). Simultaneous knockdown had an additive effect (Fig. 6B), consistent with the collaborative activity observed in co-transfection experiments (Fig. 6A). Thus, these transcription factors simultaneously contribute to *IL1B* promoter activity in HeLa cells.

We then utilized this reporter assay to determine if altered PU.1 and/or C/EBPβ transactivation could account for rs1143627 allele-specific regulation of *IL1B* gene expression. When -31 T>C alterations were introduced into the *IL1B* promoter we observed no difference in basal transcriptional activity in HeLa cells. However, upon co-transfection with PU.1, C/EBPβ, or both, the T allele produced significantly higher luciferase activity than the C variant (Fig. 6A). The degree of increased activity observed for the rs1143627 T allele was similar to that observed in primary monocytes and PBMC (Fig. 2 and Fig. 3A). Taken together, these results indicate that the SNP rs1143627 alters a *cis*-regulatory element in the *IL1B* gene that alters C/EBPβ- and PU.1-dependent expression of *IL1B* and susceptibility to TB.

## Discussion

Proinflammatory cytokines, such as IL-1β can play complex and dichotomous roles during chronic infections. This cytokine is initially required to prime the anti-mycobacterial immune response [25], [26], [45], and may also promote resistance to initial infection through the induction of epithelial antimicrobial peptides [46]. However, the prolonged production of this cytokine must be restrained to prevent chronic tissue damage [21]. Similarly complex roles for proinflammatory pathways have been described during a variety of viral and bacterial infections [47], [48], [49]. As a result, the production of these cytokines often correlates with the severity of human disease, but their complex biological activities make it difficult to determine if this expression is causing pathology or limiting it.

In this study, we define a mechanistic link between a polymorphism in the promoter of the *IL1B* gene and the severity of TB disease, which implies a causal relationship between the expression of this cytokine and the progression of pathology. A similar association between high *IL1B* expressing genotypes and inflammatory diseases has been observed in a number of previous studies. Associations between TB and *IL1B* polymorphisms at -511G>A, +3953T>C, and +3962T>C have been reported in a variety of populations [16], [20], [50], [51], [52], although our study is the first to describe a link between high Mtb-induced IL-1β production, inflammation, and TB disease. The specific *IL1B* promoter allele (rs1143627T) that was associated with TB in our large cohort was previously reported to be enriched in a smaller study of Chinese TB patients [35]. Indeed, this allele is also associated with other diseases that involve IL-1β-dependent inflammation or cell death, such as influenza [53], keratoconus [54], [55], and coronary artery disease [56]. Thus, it appears that TB susceptibility can be determined by similar mechanisms to those that underlie other inflammatory diseases of diverse etiology.

We found that a single base change in the *IL1B* promoter increased the synergistic activity of the C/EBPβ and PU.1 transcription factors leading to increased IL-1β expression in monocytes and PBMC. These two transcription factors are known to act in a coordinated manner to drive macrophage differentiation [57], and our studies suggest they also promote the effector functions of these cells. Individuals carrying the high-IL-1β-producing rs1143627T allele were more prone to develop active TB, to have severe pathology in the lung, and to harbor extrapulmonary lesions. The association between IL-1β production and severe TB disease is consistent with our recent finding that NLRP3 inflammasome dependent IL-1β enhances neutrophil recruitment and exacerbates pulmonary pathology in mice infected with Mtb [21]. In the chronically-infected mouse, IL-1β activity is restrained at a posttranslational level through the nitric oxide (NO)-dependent nitrosylation of NLRP3 [21], an essential component of the inflammasome complex that processes pro-IL-1β into its active form. As this regulatory pathway has also been described in human cells [58], the processing of rs1143627T-induced pro-IL-1β into its bioactive form is likely mediated either by residual NLRP3 activity or through inflammasome-independent processes.

Two general mechanisms could account for the TB susceptibility that is associated with increased IL-1β expression; inhibition of specific antimicrobial immunity or exacerbated inflammatory tissue damage. The former hypothesis is unlikely to explain our current observations, as IFN-γ and IL-17 responses were either unchanged or enhanced in the TB susceptible individuals. The correlation that we observed between IL-1β, IFN-γ and TB disease supports the emerging view of IFN-γ as a poor correlate of protective immunity [59]. Mtb antigen-specific IFN-γ production by Th1, multifunctional Th1, or Th1/Th17 cells, is associated with clinical severity and bacterial load in TB patients, and not protective immunity [36], [60], [61]. Instead, the significant correlation we observed between IL-1β levels and the number of neutrophils in BALF of patients with active TB supports a specific role for granulocytic inflammation in promoting the progression of TB disease. This model is consistent with the pathological role played by neutrophil recruitment in a number of TB-susceptible mouse strains [22], [62], [63], and the predominance of this cell type in the BALF of humans with active TB [29], [64].

The high IL-1β expressing rs1143627TT genotype was associated with the severity of lung pathology in TB patients both before and after anti-tuberculosis treatment. This finding is consistent with the poor treatment response of individuals with a high frequency of IL-17/IFN-γ double-producing cells [61], which our studies suggest as a possible marker of IL-1β production (Fig. 3). These observations suggest that sustained IL-1β production causes persistent lung damage that could contribute to the permanently decreased lung function observed in patients with advanced and/or recurrent TB [65]. Thus, anti-inflammatory treatments targeted to the IL-1 pathway could be a useful adjunct therapy to mitigate the long-term pulmonary impairment caused by Mtb infection, particularly for individuals of high IL-1β-expressing genotypes.

## Materials and Methods

### Ethics statement

All protocols for this study were reviewed and approved by the Research Ethics Committee of Shenzhen Third People's Hospital (No. 2012–003), and conducted according to the Declaration of Helsinki. The use, for research purposes, of excess BALF leftover from clinically indicated bronchoscopies was deemed exempt from a requirement for informed consent beyond the consent normally obtained for this clinical procedure. The Research Ethics Committee approved the collection of peripheral blood exclusively for research purposes with the written informed consent of all participants.

### Subjects and samples

Three case-control cohorts were used in this study to investigate the association between *IL1B* gene polymorphisms and susceptibility to TB. All subjects were genetically unrelated members of the Chinese Han population. The Shenzhen experimental cohort involved 1533 patients with active TB and 1445 healthy controls, which have been used in our previous *IL6* polymorphism study[10]. Of the 1533 patients, 1432 were diagnosed with pulmonary TB (PTB), and 101 were with extrapulmonanry TB (ETB) including tuberculous lymphadenitis (n = 62), tuberculous meningitis (n = 13), and osteoarticular TB (n = 26). The Shanghai cohort was used for validation and consisted of 266 PTB patients and 262 controls, which has been used for a previous genetic study on IL-17F polymorphisms and TB susceptibility [66]. The diagnosis of tuberculosis was based on clinical symptoms, radiological evidence, and findings from Mtb examination as described previously [10], [67]. Healthy controls with normal chest radiograph findings and without a clinical history of TB were recruited. The characteristics of the study population are shown in Table 1. The whole blood samples were store at −80°C after collection for DNA extraction. Peripheral blood mononuclear cells (PBMCs) were isolated from whole blood through density gradient centrifugation over Ficoll-Hypaque as described elsewhere [68] and stored in nitrogen. The broncho-alveolar lavage fluid (BALF) was collected from Mtb culture confirmed pulmonary TB patients (n = 57) before initiation of anti-TB chemotherapy.

### SNP selection and genotyping

Genomic DNA was prepared from peripheral whole blood according to the standard protocols of QIAamp DNA Blood Mini kit (Qiagen, Hilden, Germany) as described previously[10]. Since we were particularly interested in SNPs with regulatory activity, we focused on SNPs located in putative transcription factor binding sites and microRNA target sites. To search for functional SNPs, we referred to Jaspar, UniPROBE, TRANSFAC, and PITA databases, then calculated the binding score of alleles to transcription factor, or the minimum hybridization energy and thermodynamics to microRNA as described [9]. Three SNPs in the promoter (rs1143627 T>C, rs16944 G>A, rs1143623 G>C) and one in 3-UTR (rs2853550 T>C) of the *IL1B* gene were genotyped using the MassARRAY system (Sequenom, San Diego, CA) as described elsewhere [9], [10]. The relative height (intensity) of the peaks and the signal-to-noise (SNR) ratio were analyzed using Caller software to call genotypes in real-time. Typer software can apply cluster analysis to the genotype calls assigned by the Caller software. After cluster analysis, manual curation of spectra was performed to further validate the outcome. Assays with low call rates (<90%) were discarded or redesigned, i.e., all assays shown in this study have a call rate of>90%.

### HRCT examination and scoring

HRCT were performed at 10 mm section interval (120 kV, 50–450 mAs), (1 mm slice thickness, 1.5s scanning time) with a window level between 2550 and 40 Hounsfield Units (HU) and window width between 300 and 1600 HU using the Toshiba Aquilion 64 CT Scanner (Toshiba, Tokyo, Japan). HRCT scans were analyzed two independent chest radiologists and final conclusions on the findings were reached by consensus. The arbitrary scores were based on the percentage of lung parenchyma abnormality as previously described [36], [37].

### Cell preparation and cultures

Human monocytic cell lines U937 (ATCC CRL 1593) and HeLa cells (strain S3) were cultured in antibiotic-free Dulbecco's modified Eagle medium (DMEM) containing 10% fetal bovine serum (FBS) (Hyclone, Logan, UT, USA). After differentiation in the presence of phorbol 12-myristate 13-acetate (PMA, 20 ng/ml) for 24 hours, U937 cells were rested in fresh complete medium for 24 h before further stimulation. The differentiated U937 cells were exposed to heat-killed M. tuberculosis lysate (20 µg/mL), or live Mtb H37Ra strain at a multiplicity of infection of 10∶1. The cells were harvested after additional 24 hours and used for gene expression assays or nuclear protein binding activity assays.

Primary monocytes were isolated from PBMCs of 72 healthy controls (n = 24 for each rs1143627 genotype) by positive selection using magnetic CD14 MicroBeads (Miltenyi Biotech, Germany) according to the manufacturer's instructions. Purified CD14^+^ monocytes and PBMCs were transferred into a 96-well plate in serum-free AIM-V medium (Gibco, Carlsbad, CA, USA), with 2×10^5^ cells/well in the absence or presence of heat killed Mtb lysate (20 µg/mL) or Mtb 19 kDa lipoprotein (0.5 µg/mL; Lionex GmbH, Braunschweig, Germany). The cells were harvested after 24 h or 48 h and used for gene expression assays, and the supernatant was collected to determine cytokine (IL-1β, IL-1Ra, IFN-γ, and IL-17A) production using ELISA.

### Measurement of genes expression by qRT-PCR

Total RNA extraction was performed with the RNAeasy Mini kit (Qiagen, Valencia, CA), and residual DNA was digested using RNAse-free DNAse (Qiagen). cDNA was synthesized using an oligo-dT primer and SuperScript II reverse transcriptase (Invitrogen, Carlsbad, CA). Gene expression was measured using a previously described SYBR Green-based real-time quantitative PCR [69]. For all assays, target genes were normalized against the glyceraldehyde-3-phosphate dehydrogenase (*GAPDH*) level. The qPCR primers were designed as follows: for the *IL1B* gene:, 5'-TTCTTCGACACATGGGATAACG-3' (forward) and 5'-TGGAGAACACCACTTGTTGCT-3' (reverse); for the *IL1RN* gene: 5'-GGAAGATGTGCCTGTCCTGT-3' (forward) and 5'-TCTCGCTCAGGTCAGTGATG-3' (reverse); for the *PU.1* gene: 5'-CAGCTCTACCGCCACATGGA-3' (forward) and 5'-TAGGAGACCTGGTGGCCAAGA-3' (reverse); for the *C/EBPB* gene: 5'-AACTCTCTGCTTCTCCCTCTG-3' (forward) and 5'-AAGCCCGTAGGAACATCTTT-3' (reverse); for the *TBP* gene: 5'-TCTGGGATTGTACCGCAGC-3' (forward) and 5'-CGAAGTGCAATGGTCTTTAGG-3' (reverse); for the *GAPDH* gene: 5'-GCACCGTCAAGGCTGAGAAC-3' (forward) and 5'-TGGTGAAGACGCCAGTGGA-3' (reverse).

### Measurement of secreted cytokine by ELISA

The levels of IL-1β, IL-1Ra, IFN-γ, IL-17A in the supernatants of stimulated PBMCs or CD14^+^ monocytes were determined using commercially available ELISA kits (R&D, Minneapolis, MN), following the manufacturer's instructions.

### Plasmid constructs

The human *IL1B* promoter (−1292 to +79) was amplified by PCR and inserted into pGL3-Basic vector (Promega) upstream of the firefly luciferase coding region at XhoI and NcoI sites. Positive clones were subjected to site-directed mutagenesis using Quick Change Site-Directed mutagenesis Kit (Stratagene, La Jolla, CA) to obtain the desired alleles. Expression vectors for the full-length *PU.1* and *C/EBPB* were constructed by inserting the respective coding regions into pcDNA3.1 expression vector (life technology, Carlsbad, CA) at XhoI and EcoRI sites. The primer used for cloning were as follows: for *PU.1*, 5'-TATCTCGAGAACTTGTGCTGGCCCTGCAATG-3' (forward) and 5'-TATGAATTCTGTGGGGCGGGTGGCGCCGCT-3' (reverse); for *C/EBPB*, 5'-TATCTCGAGGAGTCAGAGCCGCGCACGGGACT-3' (forward) and 5'-TATGAATTCTGCAGTGGCCGGAGGAGGCGAGCAGGGGCT-3' (reverse).

### Transfection and dual-luciferase assay

HeLa cells (2×10^5^) were plated in 24-well plates 24 h before transfection. A total of 0.8 ug plasmid DNA including 0.3 ug of either rs1143627 T or rs1143627 C reporter vector, 0.2 mg of pcDNA3.1-PU.1 or pcDNA3.1-C/EBPB expression vector, and 0.1 mg of pRL-TK control vector, were co-transfected into the cells using Lipofectamine 2000 reagent (life technology). Cells were stimulated by PMA (50 ng/ml) for 20 h, following 24 h of transfection. Cells were then harvested and lysed in 150 uL passive lysis buffer (Promega), the lysates were assayed for both the firefly and Renilla luciferase activities using the dual-luciferase reporter assay system (Promega). Promoter activity was measured as the ratio between firefly and Renilla luciferase. The transfection for each construct was performed three times and each construct was assayed for promoter activity in duplicates.

### Western blotting

HeLa cells transfected with plasmid expressing PU.1 or C/EBPβ were collected 16–20 h after transfection. The cells pellet was lysed using lysis buffer (10 mM Tris, pH 7.5, 150 mM NaCl, 1% Triton X-100, 1 mM phenylmethylsulfonyl fluoride, 0.2 mM sodium orthovanadate, 0.5% Nonidet P-40) supplemented with a cocktail of protease inhibitors (Sigma-Aldrich, Steinheim, Germany). The lysates were separated by SDS-PAGE on a 12% polyacrylamide gel and then transferred onto a polyvinylidene difluoride membrane. The membranes were sequentially probed with the respective primary antibodies, followed by appropriate HRP-conjugated secondary antibodies (Promega, Madison, WI) and then visualized by exposure to X-ray films.

### Electrophoretic mobility shift assay (EMSA)

Nuclear extracts were prepared from U937 monocytes as reported previously [70]. Two sets of complementary DNA-oligonucleotide sequences containing *IL1B* rs1143627 C or T allele were designed and biotin-labelled or left unlabelled (Life technology). The oligonucleotides used were as follows: rs1143627C variant, 5'-CCTACTTCTGCTTTTGAAAGC**C**ATAAAAACAGCGA GGGAGAAA-3'; and rs1143627T variant, 5'-CCTACTTCTGCTTTTGA AAGC**T**ATAAAAACAGCGAGGAGAAA-3'. Equimolar amounts of each strand were combined in annealing buffer (10 mM Tris, 1 mM EDTA and 50 mM NaCl) by heating to 95°C for 2 min, and cooling slowly to room temperature over 2 h. EMSA assays were performed by using the LightShift chemiluminescent EMSA kit (Pierce, Rockford, IL). Binding reactions contained 20fmol biotin-labelled double- stranded probe, 2.0 uL nuclear extract and 1.0 ug poly (dI:dC) in a total volume of 20 uL binding buffer (10 mM Tris, 50 mM KCl, 3 mM MgCl2, 0.1 mM EDTA and 1.0 mM DTT). After incubation for 20 min at room temperature, complexes were separated on a 6% native polyacrylamide gel, and blotted onto a positively charged nylon membrane (Millipore, Billerica, MA), and visualized by exposure to X-ray films. EMSA images were quantified by densitometry using Quantity One, version 4.5, software (Bio-Rad). For relative quantification, the integrated optical density value was determined with background values taken below each band of interest to account for non-specific antibody staining in the lane.

For competition experiments, a 200-fold molar excess of unlabelled probe was added prior to addition of labeled probe. To assess the putative binding sites within the *IL1B* promoter in the region encompassing the SNP rs1143627, we used two bioinformatic tools, AliBaba2 and TFSearch, to predict the interaction of the different probes with proteins and set the cutoff for the dissimilarity matrix at 15%. In antibody supershift assays, anti-ZEB1, MEF2, C/EBPα, C/EBPβ, PU.1, TBP, and SP1 (each at the final concentration of 1 ug/mL, Santa Cruz Biotechnology, Santa Cruz, CA) were added after binding of nuclear extracts to labeled probe and incubated for 20 min at room temperature.

### IFN-γ ELISPOT assay

A previously established in-house IFN-γ ELISPOT assay was used to measure Mtb antigen specific IFN-γ spot forming cells (SFCs) in peripheral blood samples from patients with TB [41], [42]. Briefly, a total of 2×10^5^ cells/well of PBMCs were cultured in duplicate in 96-well plates in the presence of ESAT6 protein (protein) or peptide pools derived from ESAT6/CFP10 (peptides) for 24 h. PBMCs cultured in medium alone or in the presence of phytohemagglutinin (Sigma) at 2.5 ug/ml were used as negative or positive controls, respectively. IFN-γ producing cells were visualized as spot forming cells after incubation with biotinylated anti–IFN-γ monoclonal antibody (eBioscience) for 4 h, followed by streptavidin-alkaline phosphatase conjugate for 2 h and then substrate solution. The number of SFCs was counted using an automated ELISPOT reader (BioReader 4000 Pro-X; Biosys, Germany). The number of SFCs to protein and peptides was expressed as the number of SFCs per 0.2 million PBMCs. The background number of SFCs in the negative control well was subtracted.

### Lentiviral-mediated RNA interference

For knockdown of *PU.1*, or *C/EBPB*, we used lentiviral vectors expressing gene-specific small interfering RNA (siRNA) to specifically block expression. Oligonucleotide sequences of *PU.1* and *C/EBPB* specific siRNAs were as follows: *PU.1* siRNA, 5'-GCTTCGCCGAGAACAACTTCA-3', *C/EBPB* siRNA, 5'-TTCTCCGAACGTGTCACGTTTC-3'. The stem-loop oligonucleotides were synthesized and cloned into a lentivirus-based vector carrying the green fluorescent protein (GFP) gene (pGCSIL-GFP, Genechem, Shanghai, China). A universal sequence (LV1-NC: 5'-TTCTCCGAACGT GTCACGT-3') was used as a negative control for RNA interference. Individual human shRNA lentiviral clones were prepared and isolated as previously described [71]. For infection with siRNA-carrying lentiviral vector constructs, the viruses were diluted in serum-free OptiMEM (life technonogy) and cells were infected at a multiplicity of infection of 10 for 3 h in the presence of 5 ug/mL polybrene. After 48 h of infection in serum-containing medium, cells were harvested and tested for PU.1 or C/EBPβ expression by Western-blot.

### Statistical analysis

The Hardy-Weinberg Equilibrium (HWE) for *IL1B* polymorphisms distribution was analyzed in healthy controls and cases. The allelic and genotypic frequencies of SNPs between cases and controls were compared using the Pearson X^2^ test. The unconditional logistic regression adjusted by gender and age were performed to calculate the Odd ratios (ORs), 95% confidence intervals (CIs) and corresponding P values under four alternative models (multiplicative, additive, dominant and recessive). The one-way analysis of variance (ANOVA)/Newman-Keuls multiple comparison test was used for statistical analyses to compare the differences among multiple groups. The paired *t* test was used to compare the IFN-γ production with or without treatment of anti-IL-1β. Correlations were assessed using Spearman's rank correlation, and Pearson's *t* test was used to analyze the correlation. We used GraphPad Prism software (version 4.0) for all the statistic analysis. Two-tailed statistical tests were conducted with a significance level of 0.05.

### Accession numbers of genes mentioned in the manuscript

*IL1B*: [Homo sapiens (human)]. NCBI Gene ID: 3553.

*C/EBPB*: [Homo sapiens (human)]. NCBI Gene ID: 1051.

*PU.1*: [Homo sapiens (human)]. NCBI Gene ID: 6688.

TBP: [Homo sapiens (human)]. NCBI Gene ID: 6908.

*IL1RN*: [Homo sapiens (human)]. NCBI Gene ID: 3557.

## Supporting Information

Figure S1 Association between *IL1B* SNP and ESR/CRP levels in the peripheral blood. The ESR (A and B) and CRP (C and D) levels were determined in a total of 831 and 556 patients with pulmonary TB before initiation of anti-TB chemotherapy, respectively. The ESR levels in patients carrying different rs1143627 genotypes (A) or rs16944 genotypes (B). The CRP levels in patients carrying differentrs1143627 genotypes (C) or rs16944 genotypes (D). The differences among groups were compared using one-way ANOVA/Newman-Keuls multiple comparison test, no significant difference was found.
(TIF)

Figure S2 IL-1β promotes IFN-γ, but not IL-17A, production. PBMCs from healthy controls carrying different rs1143627 genotypes (TT, n = 24; TC, n = 24; CC, n = 24) were cultured in the absence or presence of heat killed Mtb lysate (20 µg/mL) for 48 h. The concentrations of secreted IL-1β, IFN-γ and IL-17A were determined by ELISA. Correlation analysis between the levels of IFN-γ (A) or IL-17A (C) and IL-1β was performed. The coefficient r and p value were indicated. PBMCs isolated from healthy controls were cultured in the same protocol as described above (A), without or with the addition of anti-IL1β (B) or exogenous IL-1β (D) at final concentration of 20 ug/ml. The levels IFN-γ in the supernatants were determined by ELISA. The difference of IFN-γ production between without and with treatment were compared by paired *t*-test, *P* value was indicated. * p<0.05.
(TIF)

Figure S3 Association between rs16944 SNP and Mtb-specific IFN-γ production. ESAT-6 protein (A, indicated as protein) and ESAT-6/CFP-10 peptides pool (B, indicated as peptides) specific IFN-γ production by PBMCs from patients with PTB carrying different rs16944 genotypes (AA, n = 209; AG, n = 410; and GG, n = 255) were detected by ELISPOT assay. Data were expressed the number of IFN-γ SFCs per 2×10^5^ PBMCs of each subjects. The differences among groups were compared using one-way ANOVA/Newman-Keuls multiple comparison test, no significant difference was found.
(TIF)

Table S1 Association between rs1143627 SNP and TB susceptibility in Shenzhen and Shanghai cohort.
(DOC)
