# Supplementary figures and images for: Allele-Specific Induction of IL-1β Expression by C/EBPβ and PU.1 Contributes to Increased Tuberculosis Susceptibility
Source: PLoS Pathog. 2014 Oct 16;10(10):e1004426. doi: 10.1371/journal.ppat.1004426 (PMC4199770; doi:10.1371/journal.ppat.1004426)

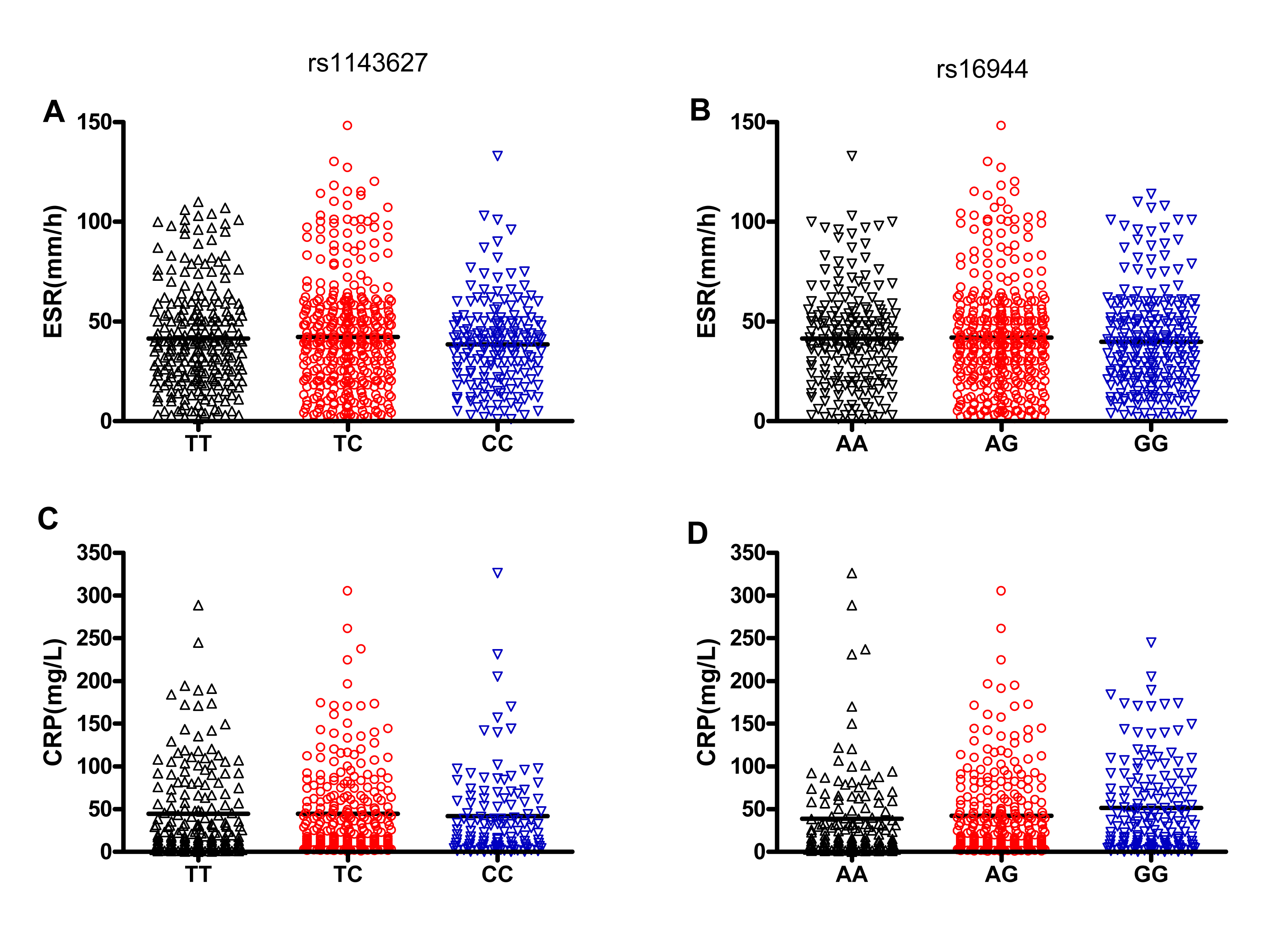

Supplement: Figure S1 — Association between IL1B SNP and ESR/CRP levels in the peripheral blood. The ESR (A and B) and CRP (C and D) levels were determined in a total of 831 and 556 patients with pulmonary TB before initiation of anti-TB chemotherapy, respectively. The ESR levels in patients carrying different rs1143627 genotypes (A) or rs16944 genotypes (B). The CRP levels in patients carrying differentrs1143627 genotypes (C) or rs16944 genotypes (D). The differences among groups were compared using one-way ANOVA/Newman-Keuls multiple comparison test, no significant difference was found. (TIF) [file ppat.1004426.s001.tif]

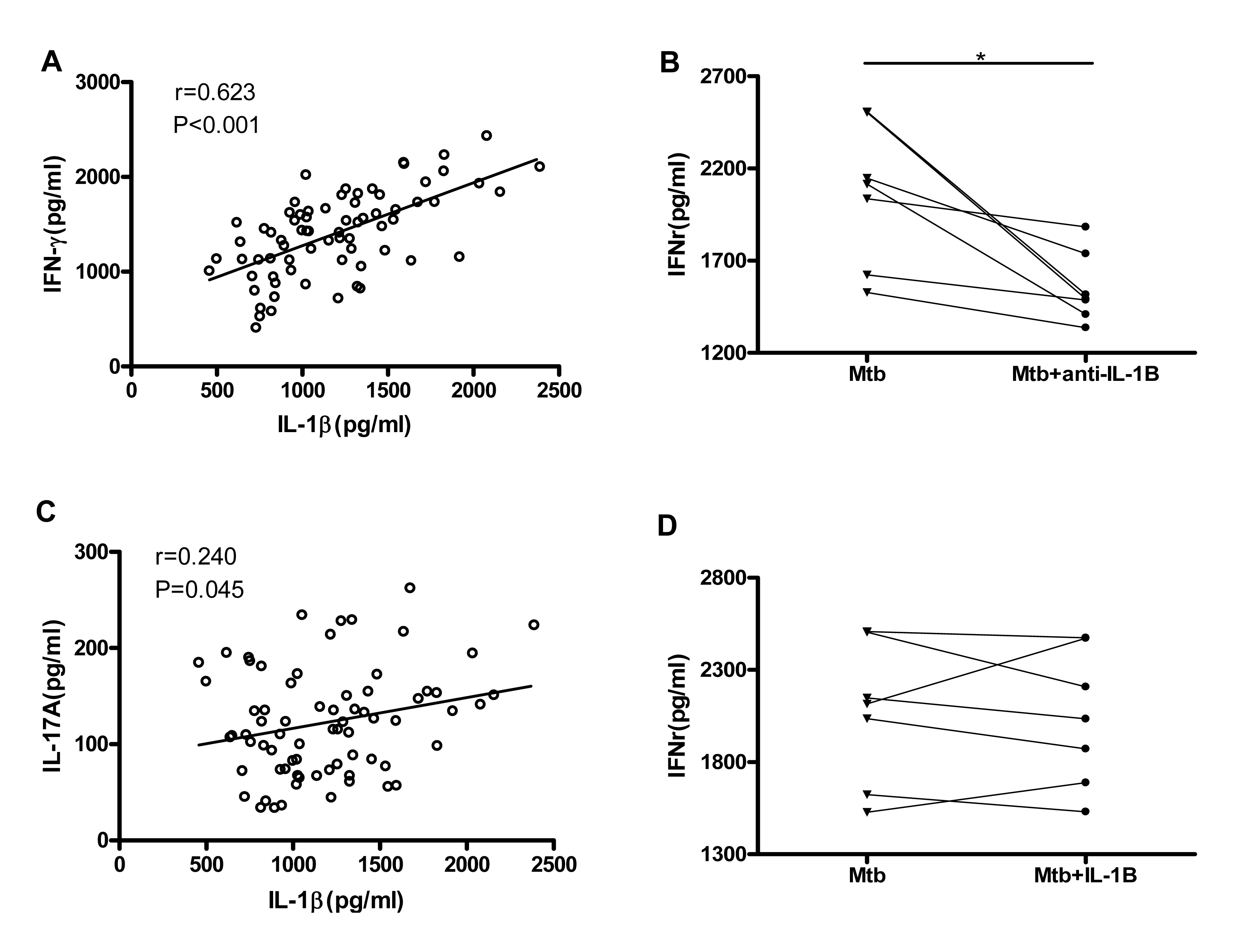

Supplement: Figure S2 — IL-1β promotes IFN-γ, but not IL-17A, production. PBMCs from healthy controls carrying different rs1143627 genotypes (TT, n = 24; TC, n = 24; CC, n = 24) were cultured in the absence or presence of heat killed Mtb lysate (20 µg/mL) for 48 h. The concentrations of secreted IL-1β, IFN-γ and IL-17A were determined by ELISA. Correlation analysis between the levels of IFN-γ (A) or IL-17A (C) and IL-1β was performed. The coefficient r and p value were indicated. PBMCs isolated from healthy controls were cultured in the same protocol as described above (A), without or with the addition of anti-IL1β (B) or exogenous IL-1β (D) at final concentration of 20 ug/ml. The levels IFN-γ in the supernatants were determined by ELISA. The difference of IFN-γ production between without and with treatment were compared by paired t-test, P value was indicated. * p<0.05. (TIF) [file ppat.1004426.s002.tif]

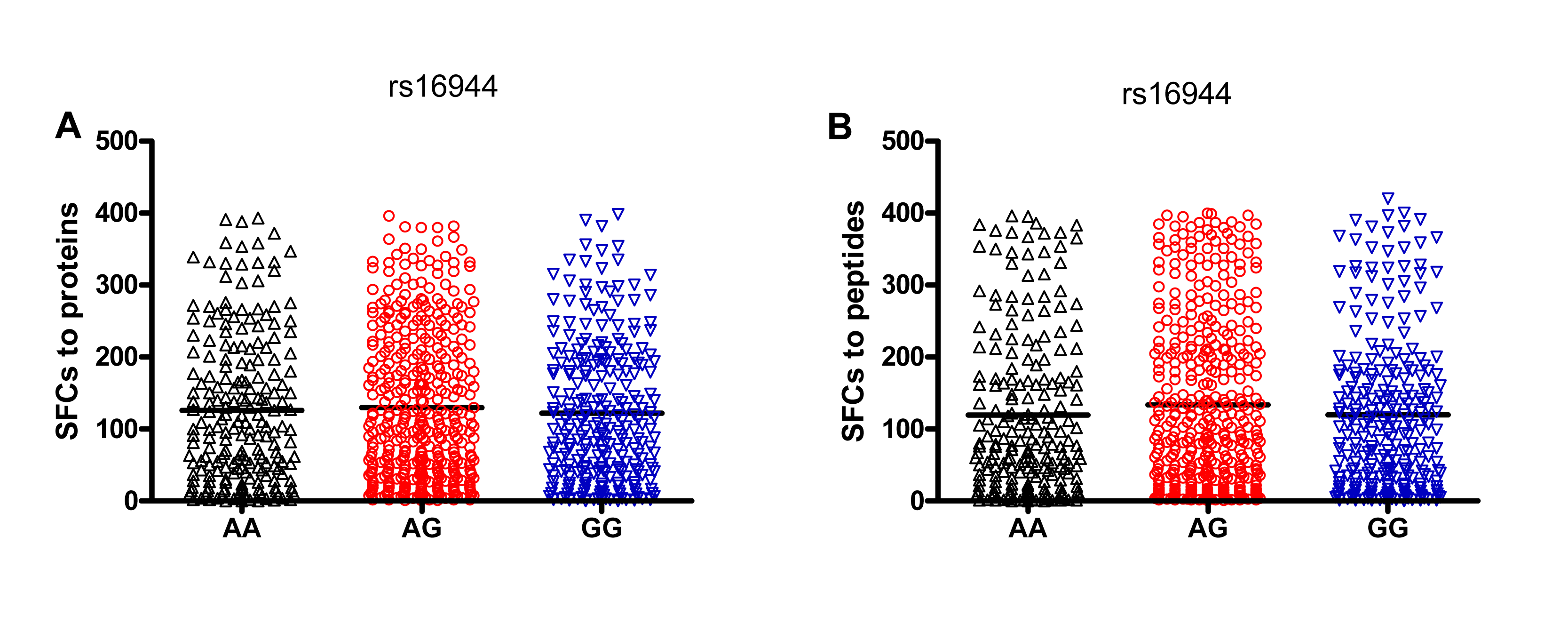

Supplement: Figure S3 — Association between rs16944 SNP and Mtb-specific IFN-γ production. ESAT-6 protein (A, indicated as protein) and ESAT-6/CFP-10 peptides pool (B, indicated as peptides) specific IFN-γ production by PBMCs from patients with PTB carrying different rs16944 genotypes (AA, n = 209; AG, n = 410; and GG, n = 255) were detected by ELISPOT assay. Data were expressed the number of IFN-γ SFCs per 2×10^5^ PBMCs of each subjects. The differences among groups were compared using one-way ANOVA/Newman-Keuls multiple comparison test, no significant difference was found. (TIF) [file ppat.1004426.s003.tif]
