# Supplementary material for: Allele-Specific Induction of IL-1β Expression by C/EBPβ and PU.1 Contributes to Increased Tuberculosis Susceptibility
Source: PLoS Pathog. 2014 Oct 16;10(10):e1004426. doi: 10.1371/journal.ppat.1004426 (PMC4199770; doi:10.1371/journal.ppat.1004426)
Supplement: Table S1 — Association between rs1143627 SNP and TB susceptibility in Shenzhen and Shanghai cohort. (DOC) [file ppat.1004426.s004.doc]

**Table S1.** Association between rs1143627 SNP and TB susceptibility in Shenzhen and Shanghai cohort

|  |  | **HC** | **TB** | **Multiplicative** | | **Additive** | | **Dominant** | | **Recessive** | |
| --- | --- | --- | --- | --- | --- | --- | --- | --- | --- | --- | --- |
| **SNP ID** | **Genotype** | **No (%)** | **No (%)** | **P Value** | **OR (95% CI)** | **P Value** | **OR (95% CI)** | **P Value** | **OR (95% CI)** | **P Value** | **OR (95% CI)** |
| rs1143627 | TT | 416 (24.4) | 517 (28.7) | <0.0001 | 1.23(1.12-1.35) | <0.0001 | 1.50(1.25-1.81) | <0.0001 | 1.38(1.18-1.61) | 0.003 | 1.25(1.07-1.45) |
|  | TC | 824 (48.3) | 897 (49.9) |  |  | 0.0009 | 1.32(1.12-1.55) |  |  |  |  |
|  | CC | 467 (27.4) | 385 (21.4) |  |  | Ref. | Ref. |  |  |  |  |

Note: Hardy-Weinberg equilibrium P values of rs1143627 were 0.16 and 0.91 in HC and TB respectively
